# Mortality in patients with acquired human immunodeficiency virus infection hospitalized in an intensive care unit during the period 2017–2019

**DOI:** 10.1038/s41598-022-19904-z

**Published:** 2022-09-19

**Authors:** Guillermo Ortiz Ruiz, Carlos Felipe López Herrera, Jorge Andrés Mahecha Bohórquez, John Edison Betancur

**Affiliations:** 1 Critical Medicine and Intensive Care and Pulmonology, Universidad del Bosque, Bogotá, Colombia; 2 National Academy of Medicine, Hospital Santa Clara, Bogotá, Colombia; 3 El Bosque University, Bogotá, Colombia; 4 Critical Medicine and Intensive Care, Hospital Santa Clara, Bogotá, Colombia

**Keywords:** Medical research, Disease prevention, Health services, Public health, Immunological disorders, Infectious diseases, Risk factors

## Abstract

Identify risk factors associated with mortality in HIV patients admitted to an ICU in the city of Bogotá. Retrospective cohort study of patients treated in an ICU during the years 2017–2019. The analysis included descriptive statistics, association tests, and a logistic regression model. A predictive model of mortality at the time of admission to the ICU was developed. 110 HIV patients were identified. Association was found between a Charlson index ≥ 6 and mortality (OR = 2.3, 95% CI 1.0–5.1) and an increase in mortality in the first 21 days of ICU stay (OR = 2.2, 95% CI 1.0–4.9). In the logistic regression analysis, the absence of highly active antiretroviral therapy (HAART) upon admission to the ICU (OR = 2.5 95% CI 1.0–6.1) and the first 21 days of ICU stay (OR = 2.3 95% CI 1.0–5.4) were associated with an increase in mortality. The predictive mortality model established that mortality was higher in patients admitted to the ICU without having previously received HAART than in those who did receive therapy at the time of admission to the ICU. In patients with HIV admitted to the ICU, the absence of HAART will negatively impact mortality during their hospital stay.

## Introduction

Starting in the 1980s with the identification of HIV as the cause of opportunistic infections at the lung level in apparently healthy patients^1^, infectious and noninfectious complications began to be seen at the metabolic and cardiovascular levels^2,3^, and respiratory compromise was the first manifestation of the disease and the most frequent cause of admission to the ICU^4,5^. After the introduction of prophylaxis against *Pneumocystis jirovecii* and the start of highly active antiretroviral therapy, the morbidity and mortality associated with HIV infection has decreased, allowing a progressive increase in the life expectancy of these patients^4^.

In the era after the introduction of antiretroviral therapy, the epidemiological profile and prognosis of HIV patients admitted to the intensive care unit changed, and severe sepsis emerged as the leading cause of mortality in the intensive care unit^6^. Mortality has been related to other factors, such as the stage of the disease and associated noninfectious comorbidities, such as cardiovascular disease, chronic kidney disease, and liver disease, due to direct injury or infection by hepatotropic viruses^5,7–10^. Among the predictors evaluated in the ICU, ventilatory support, a high APACHE score at admission (greater than 24), and the use of vasopressors are associated with mortality^5,11–13^. In contrast, early admission to the ICU is associated with a decrease in mortality^14^.

Although ICU mortality in patients with HIV has decreased, the management of these patients continues to be a challenge for the intensivist due to the systemic affection observed at the time of admission, the presence of opportunistic infections, the associated comorbidities, the social context and the antiretroviral drug interactions.

In Colombia, there are no studies of risk factors and their impact on the mortality of patients with human immunodeficiency virus infection that require management in the intensive care unit. The objective of this study was to evaluate the factors associated with mortality in patients with HIV and/or AIDS hospitalized in the intensive care unit.

## Methodology

A retrospective cohort study was carried out at Santa Clara Hospital in the city of Bogotá, which included patients older than 18 years with a diagnosis of human immunodeficiency virus (HIV) infection who were cared for in the intensive care unit (ICU) during the years 2017–2019, excluding pregnant women. The medical records of these patients were reviewed, and data variables presented by these patients at the time of admission to the ICU were extracted and entered for analysis in a matrix in Microsoft Excel previously designed by the researchers.

The outcome variable was defined as death from HIV in the ICU as a dichotomous variable (yes/no). The other variables evaluated included those related to demographic characteristics, to the diagnosis and treatment of HIV and those infections associated with the disease such as tuberculosis, the use of prophylaxis for opportunistic germs, the clinical stage of the disease, the presence of comorbidities, the type of diagnosis of admission to the ICU, the need for advanced supports, complications of ICU management, the presence and number of opportunistic infections, laboratory values and variables associated with the length of stay in the ICU. The cutoff points used to evaluate prognostic markers for HIV-1 infection were similar to those used in previous publications^15,16^.

### Statistic analysis

An analysis was carried out from descriptive and inferential statistics, in which the categorical variables are reported as proportions, the continuous variables as the mean ± SD or as median and Interquartile range (IQR) according to the type of distribution. The association between categorical variables was evaluated with the χ^2^ statistic (*p* < 0.05) and Fisher's exact test, and odds ratios were evaluated with 95% confidence intervals (95% CIs). To establish differences between the quantitative variables, the *t* test and the Mann–Whitney U test were used when appropriate, and the differences were considered statistically significant with a value of *p* < 0.05. Variables with a value of *p* < 0.1 and others considered clinically relevant were included in a logistic regression model to determine the factors associated with mortality.

By means of the formula: *P* (Y = 1) = 1/1 + exp (− α − β_1_*x*_1_ − β_2_*x*_2_ − β_3_*x*_3_), using the beta coefficients of the statistically significant variables, a predictive model of mortality was developed according to the presence or absence of the characteristics in the patients upon admission to the ICU.

All statistical analyses were performed using SPSS Statistical Software, version 25 (IBM, 2017). The study was approved by the ethics committee of Santa Clara Hospital.

### Ethics approval and consent to participate

The study was approved by the Research and Innovation Committee of the Integrated Subnetwork of Health Services Center East, State Social Entity (S.S.E.) (CIeI. 12/20) and complies with the principles of the Declaration of Helsinki. In accordance with the national regulations on retrospective studies and considering that no intervention would be carried out in humans, it was agreed with the committee of the Research and Innovation of the Integrated Subnetwork of Health Services Center East S.S.E., belonging to the health secretary of the city of Bogotá, that this study did not require informed consent from the patients.

## Results

A total of 110 patients with a diagnosis of HIV admitted to the ICU were included of which 64 (58.2%) died. The median age was 39 years (IQR 30–50). Patients older than 60 years represented 11.9% of the total. The male population represented 83.6% of the total. The majority of patients (93.6%) were initially admitted to the emergency department before admission to the ICU. A total of 65.4% of the patients had a previous HIV diagnosis upon admission to the ICU. In those patients without a previous diagnosis of HIV, the diagnosis was established in the emergency room in 18.2% of the cases and in the ICU in 12.7% of the cases. Regarding the severity of the disease, 85.5% had a clinical stage of AIDS, 88.9% had a CD4 lymphocyte count < 200 mm^3^, and 91.7% had a viral load ≥ 50 mm^3^. The use of high-intensity antiretroviral therapy before admission to the hospital was documented in 31.8% of the patients; this therapy was started in the ICU in 12.7% of the cases. Regarding comorbidities, a higher frequency of neoplasia associated with AIDS (12.8%), heart disease (6.4%) and COPD (5.5%) was observed. Forty-one percent of the patients had psychoactive substance dependence, and 44.5% of the patients had a Charlson index ≥ 6 upon admission.

Opportunistic infections were documented in 64 patients; among them, 50% had infection by one germ, 34.4% had infection by two germs and 15.6% by three germs. The main opportunistic infections identified were candidiasis (54.6%), tuberculosis (45.3%), cytomegalovirus and cryptococcosis (15%). Seventy percent of the patients were receiving prophylaxis for opportunistic infections, and 41.8% were receiving treatment for tuberculosis at the time of admission (Table 1).

**Table 1:** Bivariate analysis of mortality according to demographic, clinical and laboratory characteristics of the patients upon admission to the ICU.

Characteristic	*n* = 110 (%)	Death by HIV		*P* value*	OR	95% CI
		Yes *n* = 64 (%)	No *n* = *46* (%)			
**Demographic**						
Age: ≥ 60 years	13 (11.9)	9 (14.1)	4 (8.7)	0.552	1.7	0.4–5.9
Sex: male	92 (83.6)	52 (81.2)	40 (87)	0.427	0.6	0.2–1.8
**Disease state**						
Emergency admission	103 (93.6)	59 (92.1)	44 (95.7)	0.469	0.5	0–2.8
Previous HIV diagnosis	72 (65.4)	42 (65.6)	30 (65.2)	0.965	1	0.4–2.2
Emergency department HIV diagnosis	20 (18.2)	12 (18.7)	8 (17.4)	0.855	1	0.4–2.9
ICU HIV diagnosis	14 (12.7)	6 (9.4)	8 (17.4)	0.220	0.4	0.1–1.5
HAART therapy	35 (31.8)	16 (25)	19 (41.3)	0.072	0.4	0.2–1
HAART therapy in ICU	14 (12.7)	7 (10.9)	7 (15.2)	0.508	0.6	0.2–2.1
Tuberculosis treatment	46 (41.8)	23 (35.9)	23 (50)	0.142	0.5	0.2–1.2
Opportunistic infection prophylaxis	77 (70)	45 (70.3)	32 (69.6)	0.928	0.9	0.4–2.2
AIDS	94 (85.4)	58 (90.6)	36 (78.2)	0.077	2.6	0.8–8
CD4 lymphocyte count: < 200/mm^3,a^	64 (88.9)	34 (94.4)	30 (83.3)	0.152	3.4	0.6–18.1
Viral Load: > 50/mm^3,a^	66 (91.7)	35 (94.6)	31 (88.6)	0.366	2.2	0.3–13.1
**Comorbidities**						
Diabetes mellitus	3 (2.7)	2 (3.1)	1 (2.1)	1,000	1.4	0.1–16.5
Cardiovascular diseases	7 (6.4)	5 (7.8)	2 (4.3)	0.697	1.8	0.3–10
Chronic obstructive pulmonary disease	6 (5.4)	4 (6.2)	2 (4.3)	1,000	1.4	0.2–8.3
Cirrhosis	4 (3.6)	1 (1.6)	3 (6.5)	0.307	0.2	0–2.2
Hepatitis B	4 (3.6)	2 (3.1)	2 (4.3)	1,000	0.7	0–5.2
Psychoactive drug use	45 (41)	26 (41)	19 (41.3)	0.943	0.9	0.4–2.1
Charlson index: ≥ 6	49 (44.5)	34 (53.1)	15 (33)	0.034	23	1.0–5.1
AIDS Associated neoplasia	14 (12.8)	9 (14)	5 (10.9)	0.621	1.3	0.4–4.3
**Diagnosis of admission to ICU**						
Septic shock	69 (62.8)	40 (62.5)	29 (63)	0.954	0.9	0.4–2.1
Cardiogenic shock	3 (2.8)	1 (1.6)	2 (4.4)	0.568	0.3	0–3.8
Trauma	3 (2.8)	1 (1.6)	2 (4.3)	0.570	0.3	0–3.9
Central nervous system infection	45 (41)	30 (46.9)	15 (32.6)	0.135	1.8	0.8–4
Stroke	5 (4.6)	2 (3.1)	3 (6.5)	0.648	0.4	0–2.8
**Support requirement**						
Vassopressor support	77 (70)	43 (67.1)	34 (73.9)	0.448	0.7	0.3–1.6
Inotropic use	3 (2.8)	2 (3.1)	1 (2.1)	1,000	1.4	0.1–16.5
Ventilatory support	78 (70.9)	48 (75)	30 (65.2)	0.267	1.6	0.6–3.6
Renal support	26 (23.6)	14 (21.9)	12 (26)	0.608	0.7	0.3–1.9
Pharmacological coma	66 (61)	40 (62.5)	26 (56.5)	0.463	1.3	0.6–2.9
**Complications**						
IRIS	2 (1.8)	1 (1.6)	1 (2.2)	1,000	0.7	0–11.7
Acute renal injury	62 (56.4)	34 (53.1)	28 (60.9)	0.420	0.7	0.3–1.5
Hepatic failure	18 (16.4)	9 (14)	9 (19.6)	0.443	0.6	0.2–1.8
ARDS	5 (4.5)	2 (3.1)	3 (6.5)	0.648	0.4	0–2.8
**Opportunistic Infections**	64 (58.1)	35 (54.7)	29 (63)	0.081	0.4	0.1–1.1
Opportunistic infection: 1 agent	32 (50)	21 (60)	11 (37.9)	0.658	1.9	0.1.–33.5
Opportunistic infections: 2 agents	22 (34.4)	12 (34.2)	10 (34.4)	0.902	1.2	0–21.7
Opportunistic infections: 3 agents	10 (15.6)	2 (5.7)	8 (27.6)	0.034	0.2	0.0–5.9
**Causal agent**						
*Pneumocystis Jirovecii*	8 (12.5)	1 (2.8)	7 (24.1)	0.019	0.0	0.0–0.8
Cryptococcus	10 (15.6)	6 (17.1)	4 (13.8)	0.745	1.3	0.3–5.1
*Mycobacterium tuberculosis*	29 (45.3)	15 (42.8)	14 (48.3)	0.684	0.8	0.3–2.1
Candida	36 (54.6)	16 (45.7)	20 (69)	0.074	0.4	0.1–1
Cytomegalovirus	10 (15.6)	4 (11.4)	6 (20.7)	0.492	0.5	0.1–1.9
Histoplasma capsulatum	4 (6.2)	2 (5.7)	2 (6.9)	1.000	0.8	0.1–6.2
Hepatitis B virus	7 (10.9)	5 (14.2)	2 (6.9)	0.442	2.2	0.4–12.5
**Laboratory**						
Lactate value information	94 (85.4)	52 (55.3)	42 (44.7)			
Lactate ≥ 2	41 (43.6)	24 (46.1)	17 (40.4)	0.581	1.2	0.5–2.8
Albumin value information	57 (51.8)	28 (49.1)	29 (50.9)			
Albumin < 3.5	5 (8.7)	2 (7.1)	3 (10.3)	1,000	1.5	0.2–9.7
**Other**						
Length of stay in ICU ≤ 21 days	56 (50.9)	38 (59.3)	18 (39.1)	0.038	2.2	1.0–4.9
ICU admission-survey end, days, median (IQR)	21 (9–48)	16 (5–31)	25 (15–61)	^†^0.003		
Charlson Index, median (IQR)	6 (6–7)	7 (6–9)	6 (6–7)	^†^0.024		
Age, median (IQR)	39 (30–50)	39 (30–51)	41 (32–49)	^†^0.913		
Lactate, median (IQR)	1.8 (1.0–2.5)	1.8 (1.0–2.7)	1.8 (1.2–2.3)	^†^0.807		
Albumin, mean (SD), mean difference (SE)^§^	2.2 (0.72)	2.1 (0.77)	2.4 (0.64)	^‡^0.074	^§^0.344 (0.18)	^¶^0.03–0.72

*HIV* Human immunodeficiency virus, *ICU* intensive care unit, *HAART* Highly active antiretroviral therapy, *AIDS* Acquired immunodeficiency syndrome, *IRIS* Immune reconstitution inflammatory syndrome, *ARDS* acute respiratory distress syndrome, *IQR* interquartile range, *SD* standard deviation, *SE* standard error.

^a^Prognostic markers of HIV-1 infection.

*χ^2^ test and Fisher exact test, ^†^Mann–Whitney U test, ^‡^Independent samples τ-test. *P* < 0.05.

*OR* odds ratio; 95% CI, 95% confidence interval, ^§^Mean difference, ^¶^95% confidence interval of mean difference.

The main diagnoses for admission to the ICU were septic shock (62.8%) and central nervous system infection (41%). Serum lactate values were documented in 94 patients, in whom the median was 1.8 (IQR 1.0–2.5); among these, 45.3% had abnormal values. The serum albumin value on admission was obtained in 57 patients, and the average value was 2.2 (SD ± 0.72), with abnormal values in 8.7% of the cases.

Regarding ICU management, 70% of the patients required vasopressor support, 70.9% ventilatory support, and 61% pharmacological coma. The median time of stay of the patients in the ICU was 21 days (IQR 9–48), and 50.9% of the patients had a time of stay in the ICU equal to or less than 21 days.

The main complication during the ICU stay was acute kidney injury, which was observed in 56.4% of the cases. A total of 58.2% of the patients died during their stay in the ICU (Table 1).

In the bivariate analysis, when comparing the characteristics of the patients who died with those who did not, it was possible to show that the odds ratio (OR) for mortality was 2.3 times higher in those patients with a Charlson index ≥ 6 (95% CI 1.0–5.1). It was also evidenced that the mortality odds ratio was 2.2 times higher in the first 21 days of stay in the ICU (95% CI 1.0–4.9). A protective association was found in those patients with *P. jirovecii* infection; however, this corresponds to a type 1 error due to a low number of patients with this characteristic (Table 1).

After including in the logistic regression the variables considered statistically significant and those considered relevant in the literature, the model that presented the best fit to explain the outcome was established, finding that the odds ratio of mortality during the first 21 days of ICU stay were 2.3-fold higher (95% CI 1.0–5.4) and that the absence of highly active antiretroviral therapy at ICU admission was associated with a 2.5-fold increase in the odds ratio of mortality (95% CI 1.0–6.1) (Table 2).

**Table 2:** Logistic regression analysis of risk factors for mortality in HIV patients in the ICU.

Risk factor	OR*	*P*	95% CI
Length of stay in ICU ≤ 21 days	2.3	0.049**	1.0–5.4
HAART therapy (−)	2.5	0.037**	1.0–6.1
Charlson index ≥ 6	1.8	0.174	0.7–4.2
Age ≥ 60	1.6	0.478	0.4–6.3

*OR adjusted with length of stay in the ICU ≤ 21 days, HAART therapy (−), Charlson index ≥ 6, age ≥ 60. **p < 0.05.

Finally, a predictive model of mortality at the time of admission to the ICU was established, including those variables that maintained a statistically significant association with the outcome in the logistic regression model. Highly active antiretroviral therapy was found to be the main determinant of mortality regardless of the length of stay of patients in the ICU, achieving a decrease in mortality from 54.3 to 31.8% in those patients with an ICU stay equal to or less than 21 days and from 73.5 to 55.2% in those patients with a stay longer than 21 days (Table 3).

**Table 3:** Predictive model of mortality in HIV patients upon admission to the ICU.

HAART (−)	HAART (+)	Length of stay in ICU ≤ 21 days	Length of stay in ICU > 21 days	Death probability (%)
	**x**	**x**		31.8
	**x**		**x**	52.2
**x**		**x**		54.3
**x**			**x**	73.5

Significant values are in bold.

This decrease in mortality observed in those patients with HAART on admission to the ICU remained stable during the first 21 days of stay in the ICU and in the following days.

## Discussion

In our country, we found a high prevalence and mortality (77.2%) in patients with a diagnosis of HIV treated in highly complex hospitals^17^. Information regarding the clinical characteristics and outcomes in HIV patients admitted to the intensive care unit in our setting is scarce. A previous study carried out at the Hospital Santa Clara in Bogotá showed a mortality close to 50% in this population^18^, and the cumulative incidence of mortality in this study was 58.2%, considerably high with respect to mortality in developed countries such as the Netherlands (28.2%)^19^, Spain (25.1%)^20^, Canada (20%)^21^, and the United States (19%)^22^; however, the mortality reported in this study is comparable to that reported by Kwisera et al. in Uganda^11^.

In the era prior to HAART, ventilatory support had a close relationship with mortality in terms of pulmonary involvement by opportunistic agents, as reported by Morrison^23^; however, in this study, no relationship with mortality was evidenced. This result reflects the evolution of antiretroviral therapy and advances in the management of critical patients^24^.

The main complication observed was acute kidney injury, followed by liver failure, unlike that reported in other studies, where the main complication was respiratory failure^25^.

The proportion of elderly patients in this study was 11.9%, and a cutoff point of 60 years was considered to be associated with mortality in the multivariate analysis. This finding is consistent with the increase in mortality in HIV patients with advancing age^26^ and in the presence of comorbidities not related to this pathology^27^. This relationship is more evident with aging^28,29^.

Regardless of age, comorbidities have an impact on prognosis that can be evaluated with the Charlson index, which, depending on its value, has been associated with increased mortality in critically ill patients^30,31^. In this study, this association was present with Charlson index values ≥ 6 (OR 2.3, CI 1.0–5.1), a result similar to that reported by Vidal et al.^20^, but higher than that found in other studies where mortality increases from values of the Charlson index ≥ 3^32^.

After the onset of the HIV pandemic, one of the interventions that changed the natural history of the disease was the introduction of antiretroviral therapy^33^, significantly modifying mortality. However, the initiation of antiretroviral therapy in critically ill patients is controversial due to the possibility of adverse effects related to the presence of uncontrolled opportunistic infections, the possibility of drug interactions and changes in the pharmacodynamics of critically ill patients due to antiretroviral drugs^34^. In this study, a predictive model was carried out that showed a clear impact on mortality in critically ill patients who received antiretroviral therapy upon admission to the intensive care unit compared to those who did not receive it (31.8% vs. 54.3%). This benefit prevailed during the entire length of stay in the intensive care unit^35^.

The median length of stay of patients with HIV infection in the ICU in the era prior to the period before HAART therapy was initiated and after its introduction as standard treatment in these patients is just under 10 days^22,36^. In this study, the median length of stay was 21 days, with 49.1% of patients exceeding this ICU length of stay, which contrasts with other studies in which 11% of patients had a longer ICU stay^37^. This could be due to the presence of subacute opportunistic infections at the central nervous system level, given the number of patients who had a documented central nervous system infection at the time of admission (41%), which could contribute to the progression of central respiratory failure with the need for mechanical ventilation.

A decrease in mortality was noted from day 21 of ICU stay, which contrasts with the results of the study by Moitra et al.^38^, which demonstrated a decreased possibility of discharge home at the time of hospital discharge and an increase in mortality from day seven of ICU stay.

The increase in mortality seen in this study during the first three weeks of ICU stay could be explained by comorbidities and HIV-related patient characteristics. A high percentage of patients with AIDS (85.4%) and with CD4 lymphocyte counts < 200/mm were detected (88.9%), suggesting that this population is at high risk for AIDS-associated diseases.

It is important to emphasize that the high percentage of prophylaxis for opportunistic germs (70.6%) could explain the low isolation of *P. jirovecii*. In the case of tuberculosis as an opportunistic infection, its presence did not increase mortality when compared with other infections, which would reflect a protective effect of the treatment for this disease that patients had been receiving before admission to the ICU.

Patients with CD4 lymphocyte counts < 200 mm^3^ and viral loads ≥ 50 mm^3^ had higher mortality, although most of the patients received treatment for opportunistic infections. Unlike from what was expected, mortality was similar to that of those who did not receive prophylaxis for opportunistic germs, which suggests additional causes of mortality to the presence of this type of infection, such as those associated with sepsis of bacterial origin and their respective complications^6,39^.

This study is not without its limitations. First, it is a retrospective cohort, monocentric study, which limits the adequate collection of information and may suggest associations that could change with increasing sample size. The information was limited in data on variables that have shown an impact on mortality in previous studies, such as serum albumin values, viral load and CD4 lymphocyte count. Information on the impact of sepsis and its treatment strategies on mortality is also lacking because no information is available to compare data obtained in the days after admission with those obtained at the time of ICU admission.

A protective association was found in those patients with opportunistic infection by *P. jirovecii*, which is not plausible and is considered a result of the limited sample size; therefore, the results should be analyzed with caution. In the same way, because the analyzed data came from a single hospital institution, it is recommended that in future investigations, multicenter studies be carried out with the calculation of random and representative samples of the population.

In terms of strengths, this study represents a subpopulation of HIV patients who present to the ICU in poor general health due to severity of infection, low CD4 lymphocyte count, and lack of or low adherence to antiretroviral treatment, which is in contrast to what has been observed in the general population of HIV patients. This warrants a more comprehensive approach to finding and treating diseases that may or may not be associated with advanced stages of HIV infection.

## Conclusions

This study, which assessed risk factors for mortality in HIV patients admitted to the ICU, found that a Charlson index ≥ 6 and the first 3 weeks of ICU stay were associated with mortality; in addition, advanced age contributed to this fact. In contrast, the use of highly active antiretroviral therapy in HIV patients before admission to the ICU is associated with a decrease in mortality that is seen throughout the duration of hospitalization.

## Data Availability

The authors confirm that the data supporting the findings of this study are available within the article.

## Abbreviations

AIDS: Acquired immunodeficiency syndrome
ARDS: Acute respiratory distress syndrome
APACHE: Acute Physiology and Chronic Health Evaluation
CI: Confidence interval
COPD: Chronic Obstrucive Pulmonary Disease
HAART: Highly active antiretroviral therapy
HIV: Human immunodeficiency virus
ICU: Intensive care unit
IQR: Interquartile range
IRIS: Immune reconstitution inflammatory syndrome
SD: Standard deviation
SE: Standard error
OR: Odds ratio
